# A systematic evaluation of the quality of meta-analyses in the critical care literature

**DOI:** 10.1186/cc3803

**Published:** 2005-09-09

**Authors:** Anthony Delaney, Sean M Bagshaw, Andre Ferland, Braden Manns, Kevin B Laupland, Christopher J Doig

**Affiliations:** 1 Staff Specialist, Department of Intensive Care Medicine, Royal North Shore Hospital, Sydney, NSW, Australia; 2 Fellow, Department of Critical Care Medicine, University of Calgary, Calgary, Alberta, Canada; 3 Clinical Associate Professor, Departments of Critical Care Medicine and Medicine, University of Calgary, Calgary, Alberta, Canada; 4 Assistant Professor, Departments of Medicine and Community Health Sciences, University of Calgary, Calgary, Alberta, Canada; 5 Assistant Professor, Departments of Critical Care Medicine, Medicine and Community Health Sciences, University of Calgary, Calgary, Alberta, Canada; 6 Associate Professor, Departments of Critical Care Medicine, Medicine and Community Health Sciences, University of Calgary, Calgary, Alberta, Canada

## Abstract

**Introduction:**

Meta-analyses have been suggested to be the highest form of evidence available to clinicians to guide clinical practice in critical care. The purpose of this study was to systematically evaluate the quality of meta-analyses that address topics pertinent to critical care.

**Methods:**

To identify potentially eligible meta-analyses for inclusion, a systematic search of Medline, EMBASE and the Cochrane Database of Systematic Reviews was undertaken, using broad search terms relevant to intensive care, including: intensive care, critical care, shock, resuscitation, inotropes and mechanical ventilation. Predetermined inclusion criteria were applied to each identified meta-analysis independently by two authors. To assess report quality, the included meta-analyses were assessed using the component and overall scores from the Overview Quality Assessment Questionnaire (OQAQ). The quality of reports published before and after the publication of the QUOROM statement was compared.

**Results:**

A total of 139 reports of meta-analyses were included (kappa = 0.93). The overall quality of reports of meta-analyses was found to be poor, with an estimated mean overall OQAQ score of 3.3 (95% CI; 3.0–3.6). Only 43 (30.9%) were scored as having minimal or minor flaws (>5). We noted problems with the reporting of key characteristics of meta-analyses, such as performing a thorough literature search, avoidance of bias in the inclusion of studies and appropriately referring to the validity of the included studies. After the release of the QUOROM statement, however, an improvement in the overall quality of published meta-analyses was noted.

**Conclusion:**

The overall quality of the reports of meta-analyses available to critical care physicians is poor. Physicians should critically evaluate these studies prior to considering applying the results of these studies in their clinical practice.

## Introduction

One of the challenges that faces critical care physicians is staying up to date with the current state of knowledge, in a field that has a broad scope of practice and time dependency for many of the interventions provided. Traditional sources of information such as narrative review articles, medical textbooks and the clinical opinion of experts are often at odds with the best current available evidence [1,2]. Systematic reviews in general, and meta-analyses in particular, have been suggested as one solution to this problem [3]. Some authorities have suggested that systematic reviews and meta-analyses are the highest form of published evidence available to clinicians [4].

There are numerous incidences, however, where meta-analyses have pooled results from small trials with disparate results, and produced conflicting evidence [5-7], as well as meta-analyses that have produced results that were in conflict with the results of subsequent large randomised clinical trials (RCTs). [8-11]. When this occurs it causes difficulties for clinicians trying to apply the best available evidence in the care of their patients, as it is not clear which is the best evidence to follow. As a result, doubts have been raised about the reliability of using meta-analyses to guide clinical practice. [12-14].

If clinicians are to have confidence that the results of meta-analyses can be used to guide clinical practice, then the reports of these studies need to be of a high quality. The Overview Quality Assessment Questionnaire (OQAQ) [15] is the only validated instrument available to grade the quality of review articles [16]. It has been used to grade the quality of reports of review articles in a number of fields related to critical care. [17-19].

There were three main aims of this study. First, to describe the quality of the reports of meta-analyses that are available to critical care clinicians using the OQAQ. Second, we hypothesized that the publication of the Quality of Reporting of Meta-analyses statement (QUOROM), published in 1999 [20], that was meant to improve reporting and performance of meta-analyses, might have resulted in an improvement in the quality of meta-analyses. As such, the effect of the publication of the QUOROM statement [20] on the quality of these reports was also examined. Finally, to place the results of this assessment in a broader context, the quality of the reports of meta-analyses in the critical care literature was compared to the quality of the reports of meta-analyses and systematic reviews published in the fields of emergency medicine, anaesthesia and general surgery.

## Materials and methods

### Study sample

The search for reports of meta-analyses that addressed issues pertinent to critical care medicine was conducted using the Medline database using the PubMed interface, as well as Medline, EMBASE and the Cochrane Database of Systematic Reviews using the OVID interface. Meta-analyses were considered to be any study that statistically integrated the results of a number of primary trials, randomised clinical trials or observational studies. The search terms were individualised for each database and included terms for: critical care, critical illness, intensive care, shock, resuscitation, inotropes and mechanical ventilation. This was combined with sensitive filters to identify meta-analyses [21,22]. Searches were limited to human subjects and reports published in English. The search was limited to articles published between January 1 1994 and December 31 2003, and was completed in August 2004. Full details of the search strategy are available as an additional data file (Additional file 1).

#### Study selection

One reviewer examined the titles and abstracts of all articles returned by the search to identify potentially eligible articles. All potentially eligible studies were then retrieved and the full-text article was reviewed to determine if it met the pre-determined inclusion criteria. Assessments were conducted independently by two reviewers, with disagreements resolved by discussion, or by resort to a third reviewer if consensus could not be reached. The inclusion criteria were: the study addressed an issue pertinent to critical care medicine; study population in the included studies were adult patients; study population in the included studies were human participants; the systematic review used statistical methods to produce a summary result; the report was published in English; the report of the study was first published between 1994 and 2003.

#### Data extraction

Two reviewers independently extracted data from the included studies. Data were extracted from the reports regarding the individual components of the OQAQ, and a summary score was then determined. Within the OQAQ instrument, there are nine individual items relating to the methodological quality of the meta-analysis, including the performance of a thorough search, the avoidance of bias in the inclusion of studies, appropriately referring to the validity of the included studies, appropriately combining the results and drawing appropriate conclusions from the data. Each report was assessed as to whether it clearly met the criterion, clearly did not meet the criterion, or it partially met or it was unclear whether it had met the criterion. After assessment of each of the nine component questions, a final overall score was given, based on the answers to the previous nine questions on a scale of 1 to 7, with 7 indicating no flaws, and a score of ≥ 5 indicating that the study has only minimal or minor flaws. The full details of the OQAQ scoring questionnaire are available as Additional file 2. Data were also collected regarding the date of publication. The QUOROM statement was first published in November 1999 [20], so to allow a reasonable lag time for studies in progress or under review for publication to finish and the report to be published, those reports published prior to December 31, 2000 were adjudicated as the 'pre-QUOROM' group and those published after January 1, 2001 as the 'post-QUOROM' group. The source of the publication was also classified as to whether the publication was in a critical care journal or a journal that primarily dealt with another area of medical practice.

### Analysis

The primary analysis of the data was descriptive. The proportion of reports that met each of the criteria was determined and tabulated. The estimated mean overall quality summary score was calculated.

To assess whether the overall quality of reviews had improved after publication of the QUOROM statement, the overall quality score of reports published prior to the publication of the QUOROM statement was compared to the overall quality score of reports published after the QUOROM statement. Data from this study were compared with the data published in previous reports from the emergency medicine [17], anaesthesia [19] and general surgery [18] literature.

Agreement on the inclusion of studies was assessed using a kappa statistic. The results were summarized with means and standard deviations for normally distributed data and medians and interquartile ranges for non-normally distributed data. The means of normally distributed variables were compared using unpaired t-tests. Proportions were compared using Fisher's exact test. All statistical tests were two-sided with a p-value of < 0.05 considered significant unless otherwise stated. Statistical calculations were performed using STATA 8.2 (College Station, TX, USA).

## Results

### Search results

**Figure 1:**
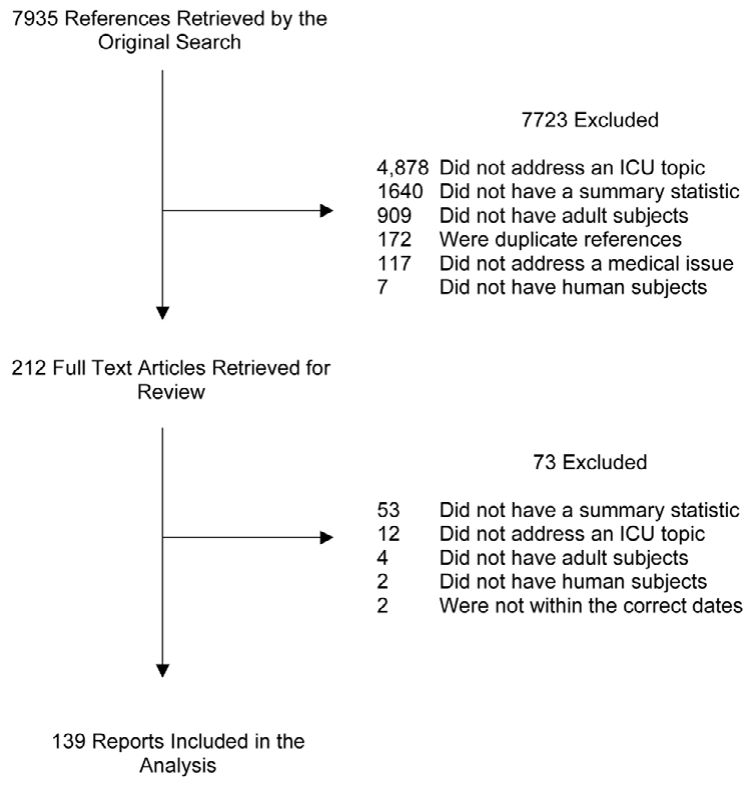
Flow chart showing results of search and reasons for exclusion of reports. ICU, intensive care unit.

A total of 7,935 articles were returned by the initial search. Of these 7,723 were deemed ineligible after inspection of the titles and abstracts. A total of 212 unique reports were retrieved for further review, and 139 were considered to be eligible for inclusion. Agreement on the inclusion of articles occurred in 97.8% of cases, which gave a kappa = 0.93 (p < 0.0005). A wide range of topics were addressed by the meta-analyses, the most common of which are shown in Table 1. A full list of the references is available as Additional file 3. The reasons for exclusion of reports, and the flow of studies are shown in Fig. 1. Table 2 shows the source of publication of the reports. The reports of meta-analyses were published in a wide variety of sources, with the majority of reports being published in sources that were not classified as critical care journals.

### The overall quality meta-analyses in the critical care literature

Agreement was reached on the scoring of all component scores and the overall quality scores without the need for resort to a third reviewer. Table 3 contains the summary results of the quality assessment of all meta-analyses that addressed topics relevant to critical care. The results for each individual study are shown in Additional file 4. Of note is that the weakest areas within the included meta-analyses were the failure to report whether a comprehensive literature search was conducted and failure to report how bias in the inclusion of studies was avoided, with only 35.3% of reports adequately fulfilling these criteria. Less than half of the reports referred to the validity of the included studies by appropriate criteria in the text.

The overall quality scores are shown in Table 4. The estimated mean overall quality score for meta-analyses published in the critical care literature from 1994 to 2003 was 3.3 (95% CI; 3.0–3.6). A total of 43 (30.9%) reports had minimal or minor flaws as shown by an overall score of ≥ 5, and 96 (69.1%) reports had major or extensive flaws, scoring ≤ 4 on the overall quality summary score.

### Has the quality of meta-analyses in the critical care literature improved over time?

**Figure 2:**
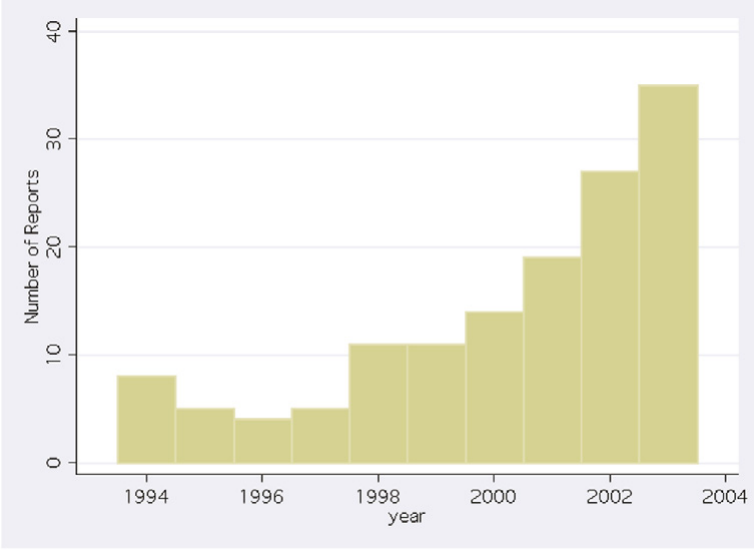
Frequency histogram showing the number of reports of meta-analyses addressing critical care issues per year, 1994 to 2003.

An increasing number of reports of meta-analyses were published in the later years of the study (Fig. 2). There were 59 reports of meta-analyses published on or before December 31 2000 that were classified as 'pre-QUOROM' and 80 reports of meta-analyses published on or after January 1 2001 that were classified as 'post-QUOROM'. Table 5 shows the number and proportion of reports that clearly fulfilled each of the components of the OQAQ (i.e. scored 'yes'). The failure to refer to the validity of the included studies occurred in 39% and 52.5% of reports pre- and post-QUOROM, respectively (p = 0.13 Fishers's exact test). All other components showed a significant improvement after the publication of the QUOROM statement.

The estimated mean quality score of the reports was 2.8 (95% CI; 2.3–3.2), and 3.7 (95% CI; 3.3–4.1) pre- and post-QUOROM, respectively. This represented an estimated improvement of 0.96 (95% CI; 0.4–1.6, p = 0.0018 two sided t-test).

### Comparison of the quality of meta-analyses in the critical care literature and in the emergency medicine, anaesthesia and general surgical literature

Three previous published studies have assessed the quality of reports of meta-analyses in the emergency medicine, anaesthesia, and general surgery fields. These studies included 29 reports of meta-analyses published in five emergency medicine journals from 1988 to 1998. [17], 82 reports of meta-analyses that addressed issues pertinent to anaesthesia identified up until June 1999, from a Medline search not limited solely to anaesthesia journals [19], and 51 meta-analyses that addressed general surgery issues from 1997 to 2002 [18]. The estimates of the mean overall quality scores for the emergency medicine, anaesthesia, general surgery and critical care, as well as the estimates of the proportions of reports that had minimal or minor flaws only (i.e. had scored ≥ 5 on the OQAQ overall quality score) are shown in Table 6. It should be noted that the overall quality of reports was poor for each discipline, with the estimated mean OQAQ scores being <5 in each discipline and with less than 50% of all reports having a score of ≥ 5 in each discipline.

## Discussion

Many reports of meta-analyses address topics pertinent to critical care available to physicians. The number of reports is increasing with time, as has been demonstrated in a number of other studies [19,23]. If critical care physicians are to use these reports to guide their clinical practice, they cannot rely on browsing solely from critical care journals, as the majority of reports of meta-analyses are not published in critical care journals. The result of this study raises questions about the quality of those reports, however, and therefore whether they can be recommended without qualification as the best evidence to guide clinical practice at the present time.

It was found that the overall quality of reports of meta-analyses in addressing critical care topics is generally poor. Studies with an overall OQAQ score of 5 or more are regarded as having minimal or minor flaws. The average score of the reports in the critical care literature was only 3.3, so clearly the majority of reports are of an inferior quality. Less than one-third of reports had a score of 5 or more. This places an important caveat on the recommendation that these reports are the highest quality evidence available. Clinicians must still critically appraise the reports prior to consideration of the recommendations made in the report of the meta-analysis [4].

While the overall quality of reports is of some interest, the results of the component scores of the OQAQ may offer more insight into the areas that should be improved. The areas that were most poorly attended to were the conduct of a comprehensive search, the avoidance of bias in the selection of studies and the assessment of the validity of all the included studies. These are crucial elements in the conduct of a meta-analysis, without which the results of the meta-analysis will be questionable. Authors contemplating conducting meta-analyses and reviewers assessing studies for publication may be able to focus on these aspects of the conduct and reporting of meta-analyses in order to have the greatest impact on improving their overall quality.

There is some cause for optimism, however. Clearly the quality of reports of meta-analyses has improved over time. While it is hard to pinpoint the exact cause of the improvement, it may be that the dissemination of guidelines such as the QUOROM statement [20] has been associated with an improvement in the quality of reports. A similar improvement in the quality of reports has been found with regards to the quality of reports of RCTs following the publication of the Consolidated Standards of Reporting Trials (CONSORT) statement [24]. It is also possible that increased attention paid to the general methodological quality of reports by journal editors and reviewers has also played a role. Both of these factors may be contributing to a general global trend for better quality research. Authors, reviewers and journal editors should be encouraged to follow these guidelines in the hope that a more standard, high quality report of this type of study will become the norm, and clinicians can spend more time considering the results of the meta-analysis, rather than scrutinizing the methodological quality of the report.

It was found that the quality of the meta-analyses in the critical care literature was comparable to the quality of reviews published in the emergency medicine [17], the anaesthesia [19] and the general surgery literature [18]. There were some differences in the conduct of this study compared to the conduct of the previous studies that makes comparing the results somewhat problematic. While this makes it difficult to draw strong conclusions regarding the comparative quality of the reviews in the different fields, the lower quality of the scores in the emergency medicine literature may reflect the temporal trend seen in the critical care literature. The slightly higher scores in the anaesthesia literature may reflect differences in implementation of the scoring system. It should suffice to note that there is ample room for improvement in the quality of the reviews in each of the fields.

There are a number of limitations to this study. Critical care is an area of medicine that covers a wide variety of fields. As such, sampling the meta-analyses that address critical care topics is difficult. While attempts were made to include a diverse range of search terms, it is possible that some studies were not identified by the search strategy employed in this study. The studies not included could have different characteristics to those included, although it is unlikely that they are systematically different. It is also important to note that while the OQAQ is the instrument most widely used to grade the quality of meta-analyses and systematic reviews, it has not had extensive validation testing, nor validation testing since the establishment of the QUORUM guidelines [16].

While it would be hoped that high quality meta-analyses would produce the results that are concordant with the results of other high quality evidence, such as well-conducted, large RCTs, this is not necessarily the case. Due to differences in the interventions tested, populations, outcomes measured and other confounding issues, it is difficult to determine when meta-analyses will agree with RCTs that address the same issue. Previous studies [12,25,26] that have examined the relationship between the results of the meta-analyses and large RCTs have not addressed the issue of the methodological quality of the meta-analyses or the RCTs, another issue that may confound this relationship. Uncertainty about when the results of meta-analyses can be used to guide clinical practice rather than a future research agenda remains and further methodological investigation in this area is still needed.

## Conclusion

A large number of reports of meta-analyses address issues pertinent to critical care, and these numbers are increasing over time. These reports appear in a wide variety of sources. Physicians wishing to use the results of these studies to guide their clinical practice would need to employ strategies other than browsing critical care journals in order to access all the relevant reports. The overall quality of the reports is low, and the majority of reports of meta-analyses are not of a methodological quality whereby the results of the study could be reliably used to guide clinical practice. There is, however, some hope that improvement in the quality of the reports subsequent to the publication of the QUOROM guidelines will continue, and authors and reviewers should be encouraged to follow established methodological guidelines for the conduct and reporting of these studies.

## Key messages

• 	The overall quality of meta-analyses that address topics pertinent to critical care medicine is poor.

• 	Meta-analyses need to be critically appraised prior to the results being considered applicable to guide clinical practice.

• 	The main areas that were reported to be deficient were the conduct of a reasonably thorough search, the avoidance of bias in the inclusion of studies and referring to the validity of the included studies appropriately. Authors should pay greater attention to these aspects of the meta-analytic process in the conduct and reporting of their study.

• 	Authors, reviewers and journal editors could improve the reporting of meta-analyses by more closely adhering to the established methodological guidelines such as the QUOROM statement.

## Abbreviations

OQAQ = Overview Quality Assessment Questionnaire; QUOROM = Quality of Reporting of Meta-analyses; RCT = randomised clinical trial.

## Competing interests

This work was part of a thesis submitted to the faculty of graduate studies in partial fulfilment of the requirement for the degree of Master of Science, Department of Community Health Science, University of Calgary.

## Authors' contributions

AD and CD conceived the study. AD was responsible for the design of the study, searching for studies, selection of studies, data acquisition and analysis. CD was responsible for the design of the study, selection of studies and data acquisition. SB was responsible for the selection of studies. AF, BM and KL were all involved in the design of the study. All authors were involved in the drafting of the manuscript and gave approval of the final version.

## Supplementary Material

Additional File 1 Word file (doc) providing full details of the search strategy to Identify Meta-analyses pertinent to Critical Care Medicine. Click here for file

Additional File 2 Word file (doc) providing full details of the OQAQ scoring questionnaire. Click here for file

Additional File 3 Word file (doc) providing a full list of the references used in this study. Click here for file

Additional File 4 Spreadsheet (xls) listing the quality assessment results for each individual meta-analysis included in this study. Click here for file

## Figures and Tables

**Table 1:** Common topics addressed by meta-analyses in the critical care literature

Topic	Number of reports
Nutrition	13
Fluid therapy	11
Central venous catheters	10
Traumatic brain injury	10
Variceal bleeding	9
Non-invasive ventilation	8
Selective decontamination	7
Oxygen delivery	6
Intervention in sepsis and septic shock	6
Cardiac arrest	5
Therapy for acute renal failure	4
Blood transfusion	3
Sedation	3
Low tidal volume ventilation	2
Eclampsia	2
Nitric oxide	2
Deep Venous Thrombosis prophylaxis	2
Heliox for acute asthma	2
Stress ulcer prophylaxis	2
Other issues	32

**Table 2:** Source of publication of reports of meta-analyses that address critical care issues

Source of publication	Number of reports	Percentage
Cochrane database of systematic reviews	37	26.6%
Critical care journals	36	25.9%
Specialty medicine journals	29	20.9%
General medicine journals	15	10.8%
Anaesthesia journals	5	3.6%
General surgery journals	5	3.6%
Nursing journals	3	2.2%
Specialty surgery journals	1	0.7%
Other journals	8	5.8%

**Table 3:** Overview Quality Assessment Questionnaire component score results

OQAQ question	No (n (%))	Partial or can't tell (n(%))	Yes (n(%))
Were the search methods used to find evidence on the primary question(s) stated	5 (3.6)	3 (2.2)	131 (94.2)
Was the search for evidence reasonably comprehensive?	23 (16.6)	67 (48.2)	49 (35.3)
Were the criteria used for deciding which studies to include in the overview reported?	14 (10.1)	7 (5.0)	118 (84.9)
Was bias in the selection of studies avoided?	27 (19.4)	63 (45.3)	49 (35.3)
Were the criteria used for assessing the validity of the included studies reported?	38 (27.3)	8 (5.8)	93 (66.9)
Was the validity of all the studies referred to in the text assessed using appropriate criteria?	45 (32.4)	29 (20.9)	65 (46.8)
Were the methods used to combine the findings of the relevant (to reach a conclusion) reported?	12 (8.6)	17 (12.2)	110 (79.1)
Were the findings of the relevant studies combined appropriately relative to the primary question of the overview?	14 (10.1)	37 (26.6)	88 (63.3)
Were the conclusions made by the author(s) supported by the data and/or analysis reported in the overview?	6 (4.3)	29 (20.9)	104 (74.8)

Data expressed as total number of reports with that score (percent).

**Table 4:** Overview Quality Assessment Questionnaire summary score results

Overall OQAQ score	n (%)
1	26 (18.7)
2	37 (26.6)
3	10 (7.2)
4	23 (16.6)
5	26 (18.7)
6	10 (7.2)
7	7 (5.0)

Data expressed as total number of reports receiving that score (percent).

**Table 5:** Comparison of reports that fulfilled each OQAQ component pre-QUOROM and post-QUOROM

OQAQ question	Pre-QUOROM (n (%))	Post-QUOROM (n (%))	p-value
Were the search methods used to find evidence on the primary question(s) stated	52 (88.1)	79 (98.8)	0.010
Was the search for evidence reasonably comprehensive?	14 (23.7)	35 (43.8)	0.019
Were the criteria used for deciding which studies to include in the overview reported?	44 (74.6)	74 (92.5)	0.007
Was bias in the selection of studies avoided?	15 (25.4)	34 (42.5)	0.048
Were the criteria used for assessing the validity of the included studies reported?	33 (55.9)	60 (75.0)	0.028
Was the validity of all the studies referred to in the text assessed using appropriate criteria?	23 (39.0)	42 (52.5)	0.13
Were the methods used to combine the findings of the relevant (to reach a conclusion) reported?	40 (67.8)	70 (87.5)	0.006
Were the findings of the relevant studies combined appropriately relative to the primary question of the overview?	29 (49.2)	59 (73.8)	0.004
Were the conclusions made by the author(s) supported by the data and/or analysis reported in the overview?	35 (59.3)	69 (86.3)0	<0.0005

Data expressed as the number of reports that scored 'yes' for each component (percent). P-values derived from Fisher's exact test. OQAQ, Overview Quality Assessment Questionnaire; QUOROM, Quality of Reporting of Meta-analyses.

**Table 6:** Comparison of the overall quality of reports of meta-analyses in the emergency medicine, anaesthesia and critical care literature

	Emergency medicine	Anaesthesia	General surgery	Critical care
Mean overall OQAQ score (95% CI)	2.7 (2.1–3.2)	4.3 (3.8–4.7)	3.3 (2.8–3.9)	3.3 (3.0–3.6)
Proportion of reports with an overall OQAQ score ≥5 (95% CI)	13.8 (3.9–31.6)	41.5 (30.7–52.9)	25.5 (14.3–39.6)	30.9 (23.4–39.3)
