# Supplementary material for: A systematic evaluation of the quality of meta-analyses in the critical care literature
Source: Crit Care. 2005 Sep 9;9(5):R575–82. doi: 10.1186/cc3803 (PMC1297628; doi:10.1186/cc3803)
Supplement: Additional File 1 — Word file (doc) providing full details of the search strategy to Identify Meta-analyses pertinent to Critical Care Medicine. [file cc3803-S1.doc]

**Search Strategy to Identify Meta-analyses pertinent to Critical Care Medicine.**

1. PubMed MEDLINE Search

- (*critical* OR intensive* OR intensive care OR intensive care units OR "intensive therapy" OR critically ill OR critical illness OR critical care)* OR
- (*cardiotonic agents OR sympathomimetic OR vasoconstrictor agents OR artificial respiration OR mechanical ventilation OR resuscitation OR shock OR multiple organ failure*)
  AND
- *(((meta-analysis [pt] OR meta-analysis [tw] OR metanalysis [tw]) OR ((review [pt] OR guideline [pt] OR consensus [ti] OR guideline* [ti] OR literature [ti] OR overview [ti] OR review [ti]) AND ((Cochrane [tw] OR Medline [tw] OR CINAHL [tw] OR (National [tw] AND Library [tw])) OR (handsearch* [tw] OR search* [tw] OR searching [tw]) AND (hand [tw] OR manual [tw] OR electronic [tw] OR bibliographi* [tw] OR database* OR (Cochrane [tw] OR Medline [tw] OR CINAHL [tw] OR (National [tw] AND Library [tw]))))) OR ((synthesis [ti] OR overview [ti] OR review [ti] OR survey [ti]) AND (systematic [ti] OR critical [ti] OR methodologic [ti] OR quantitative [ti] OR qualitative [ti] OR literature [ti] OR evidence [ti] OR evidence-based [ti]))) BUTNOT (case* [ti] OR report [ti] OR editorial [pt] OR comment [pt] OR letter [pt])[1]*

2. OVID EMBASE, MEDLINE and Cochrane Database of Systematic Reviews searches.

An initial search for articles pertinent to critical care was run in all three databases using the strategy:

*1. intensive care.mp. or exp Intensive Care/*

*2. critical care.mp. or exp Critical Care/*

*3. critical illness.mp. or exp Critical Illness/*

*4. 1 or 2 or 3*

AND a sensitive filter to identify meta-analyses[2]

*1. meta-analysis.pt.*

*2. meta-anal:.tw.*

*3. metaanal:.tw.*

*4. quantitativ: review:.tw.*

*5. quantitativ: overview:.tw.*

*6. systematic: review:.tw.*

*7. systematic: overview:.tw.*

*8. methodologic: review:.tw.*

*9. methodologic: overview:.tw.*

*10. review.pt.*

*11. medline:.tw.*

*12. 10 and 11*

*13. 2 or 3 or 4 or 5 or 6 or 7 or 8 or 9 or 12*

This search was supplemented by a search of EMBASE using the terms

*1. exp TRAUMATIC SHOCK/ or exp HYPOVOLEMIC SHOCK/ or exp BURN SHOCK/ or shock.mp. or exp ANAPHYLACTIC SHOCK/ or exp SHOCK/ or exp HEMORRHAGIC SHOCK/ or exp SHOCK LUNG/ or exp SEPTIC SHOCK/ or exp CARDIOGENIC SHOCK/*

*2. resuscitation.mp. or exp RESUSCITATION/*

*3. multiple organ failure.mp. or exp Multiple Organ Failure/*

*4. exp Noradrenalin/ or exp Dobutamine/ or exp Inotropic Agent/ or inotrope.mp. or exp Adrenalin/ or exp Dopamine/*

*5. mechanical ventilation.mp. or exp Artificial Ventilation/*

Again combined with the filter to identify meta-analyses[2]

The final search was of the Cochrane Database of Systematic Reviews using the search strategy

*1. resuscitation.mp. [mp=title, short title, abstract, full text, keywords, caption text]*

*2. mechanical ventilation.mp. [mp=title, short title, abstract, full text, keywords, caption text]*

*3. artificial respiration.mp. [mp=title, short title, abstract, full text, keywords, caption text]*

*4. inotrope.mp. [mp=title, short title, abstract, full text, keywords, caption text]*

*5. shock.mp. [mp=title, short title, abstract, full text, keywords, caption text]*

*6. multiple organ failure.mp. [mp=title, short title, abstract, full text, keywords, caption text]*

*7. 1 or 2 or 3 or 4 or 5 or 6*

*8. limit 7 to systematic reviews*

Searches were limited to articles published in English and dealing with human subjects published between January 1, 1994 and December 31, 2003.

References

1. Shojania KG, Bero LA: **Taking advantage of the explosion of systematic reviews: an efficient MEDLINE search strategy**. *Eff Clin Pract* 2001, **4**(4):157-162.

2. Hunt DL, McKibbon KA: **Locating and appraising systematic reviews**. *Ann Intern Med* 1997, **126**(7):532-538.
