# Supplementary material for: A systematic evaluation of the quality of meta-analyses in the critical care literature
Source: Crit Care. 2005 Sep 9;9(5):R575–82. doi: 10.1186/cc3803 (PMC1297628; doi:10.1186/cc3803)
Supplement: Additional File 2 — Word file (doc) providing full details of the OQAQ scoring questionnaire. [file cc3803-S2.doc]

**Data Collection Form For the Quality of Meta-analyses in the Critical Care Literature**

Reference:

|  | Quality Features | 1 | 2 | 3 |
| --- | --- | --- | --- | --- |
| 1 | Were the Search methods used to find evidence on the primary question(s) stated |  |  |  |
| 2 | Was the search for evidence reasonably comprehensive? |  |  |  |
| 3 | Were the criteria used for deciding which studies to include in the overview reported? |  |  |  |
| 4 | Was bias in the selection of studies avoided? |  |  |  |
| 5 | Were the criteria used for assessing the validity of the included studies reported? |  |  |  |
| 6 | Was the validity of all the studies referred to in the text assessed using appropriate criteria? |  |  |  |
| 7 | Were the methods used to combine the findings of the relevant (to reach a conclusion) reported? |  |  |  |
| 8 | Were the findings of the relevant studies combined appropriately relative to the primary question of the overview? |  |  |  |
| 9 | Were the conclusions made by the author(s) supported by the data and/or analysis reported in the overview? |  |  |  |

10. How would you rate the quality of this overview?

| Flaws | | | | | | | |
| --- | --- | --- | --- | --- | --- | --- | --- |
| Extensive | |  | | Minor | | |  |
|  | Major | | | |  | Minimal | |
| 1 | 2 | 3 | 4 | | 5 | 6 | 7 |

# Quality Features:

1 = No

2 = Partially or Can’t tell

3 = Yes

If the methods that were used are reported incompletely relative to a specific item, score that item as “partially.” Similarly, if there is no information provided regarding what was done relative to a particular question, score it as “can’t tell,” unless there is information in the overview to suggest either that the criterion was or was not met.

For Question 2, for a search to be considered comprehensive the methods used to perform the search should include searching for unpublished material as well as multiple medical databases (EMBASE as well as MEDLINE). If only published material was searched for the search should be marked “partially”

For Question 4, for bias to have been avoided in the selection of studies, the report should indicate that explicit criteria were used to define studies eligible for inclusion.

For question 6, to determine whether the validity was assessed using appropriate criteria, all the studies in the text must have had their validity assessed and explicit criteria which were appropriate for the type of research question that was being addressed must have been used.

For Question 8, if no attempt has been made to combine findings, and no statement is made regarding the inappropriateness of combining findings, check “no.” If a summary (general) estimate is given anywhere in the abstract, the discussion, or the summary section of the paper, and it is not reported how that estimate was derived, mark “no,” even if there is a statement regarding the limitations of combining the findings of the studies reviewed. If in doubt, mark “can’t tell.”

For an overview to be scored as “yes” on Question 9, data (not just citations) must be reported that support the main conclusions regarding the primary question(s) that the overview addresses.

The score for Question 10, the overall scientific quality, should be based on your answers to the first 9 questions. The following guidelines can be used to assist with deriving a summary score: if the “can’t tell” option is used one or more times on the preceding questions, a review is likely to have minor flaws at best, and it is difficult to rule out major flows (ie, a score ≤4). If the “no” option is used on Questions 2, 4, 6, or 8, the review is likely to have major flaws (ie, a score of ≤3, depending on the number and degree of the flaws).
