# Supplementary material for: A systematic evaluation of the quality of meta-analyses in the critical care literature
Source: Crit Care. 2005 Sep 9;9(5):R575–82. doi: 10.1186/cc3803 (PMC1297628; doi:10.1186/cc3803)
Supplement: Additional File 3 — Word file (doc) providing a full list of the references used in this study. [file cc3803-S3.doc]

**Meta-analyses that address topics pertinent to Critical Care Medicine**

**1994-2003.**

1. Al-Omran M, Groof A, Wilke D: **Enteral versus parenteral nutrition for acute pancreatitis**. *Cochrane Database of Systematic Reviews* 2004, **2**:2.

2. Alderson P, Bunn F, Lefebvre C, Li WP, Li L, Roberts I, Schierhout G: **Human albumin solution for resuscitation and volume expansion in critically ill patients.[see comment][update of Cochrane Database Syst Rev. 2000;(2):CD001208; PMID: 10796756]**. *Cochrane Database of Systematic Reviews* 2002(1):CD001208.

3. Alderson P, Roberts I: **Corticosteroids for acute traumatic brain injury**. *Cochrane Database Syst Rev* 2000(2):CD000196.

4. Alderson P, Schierhout G, Roberts I, Bunn F: **Colloids versus crystalloids for fluid resuscitation in critically ill patients**. *Cochrane Database Syst Rev* 2000(2):CD000567.

5. Alejandria MM, Lansang MA, Dans LF, Mantaring JBV: **Intravenous immunoglobulin for treating sepsis and septic shock**. *Cochrane Database of Systematic Reviews* 2004, **2**:2.

6. Annane D, Bellissant E, Bollaert PE, Briegel J, Keh D, Kupfer Y: **Corticosteroids for treating severe sepsis and septic shock**. *Cochrane Database Syst Rev* 2004(1):CD002243.

7. Ariano RE, Kassum DA, Aronson KJ: **Comparison of sedative recovery time after midazolam versus diazepam administration**. *Crit Care Med* 1994, **22**(9):1492-1496.

8. Attia J, Ray JG, Cook DJ, Douketis J, Ginsberg JS, Geerts WH: **Deep vein thrombosis and its prevention in critically ill adults**. *Arch Intern Med* 2001, **161**(10):1268-1279.

9. Banares R, Albillos A, Rincon D, Alonso S, Gonzalez M, Ruiz-del-Arbol L, Salcedo M, Molinero LM: **Endoscopic treatment versus endoscopic plus pharmacologic treatment for acute variceal bleeding: a meta-analysis**. *Hepatology* 2002, **35**(3):609-615.

10. Beale RJ, Bryg DJ, Bihari DJ: **Immunonutrition in the critically ill: a systematic review of clinical outcome**. *Crit Care Med* 1999, **27**(12):2799-2805.

11. Biondi-Zoccai GGL, Abbate A, Parisi Q, Agostoni P, Burzotta F, Sandroni C, Zardini P, Biasucci LM: **Is vasopressin superior to adrenaline or placebo in the management of cardiac arrest? A meta-analysis**. *Resuscitation* 2003, **59**(2):221-224.

12. Boeuf B, Gauvin F, Guerguerian AM, Farrell CA, Lacroix J, Jenicek M: **Therapy of shock with naloxone: a meta-analysis**. *Crit Care Med* 1998, **26**(11):1910-1916.

13. Boeuf B, Poirier V, Gauvin F, Guerguerian AM, Roy C, Farrell CA, Lacroix J: **Naloxone for shock**. *Cochrane Database of Systematic Reviews* 2004, **2**:2.

14. Boyd O: **Peri-operative cardiovascular optimization**. *Bailliere's Best Practice in Clinical Anaesthesiology* 1999, **13**(3):267-277.

15. Boyd O: **Optimisation of oxygenation and tissue perfusion in surgical patients**. *Intensive & Critical Care Nursing* 2003, **19**(3):171-181.

16. Braunschweig CL, Levy P, Sheean PM, Wang X: **Enteral compared with parenteral nutrition: A meta-analysis**. *American Journal of Clinical Nutrition* 2001, **74**(4):534-542.

17. Bunn F, Alderson P, Hawkins V: **Colloid solutions for fluid resuscitation**. *Cochrane Database Syst Rev* 2003(1):CD001319.

18. Bunn F, Roberts I, Tasker R, Akpa E: **Hypertonic versus isotonic crystalloid for fluid resuscitation in critically ill patients**. *Cochrane Database Syst Rev* 2002(1):CD002045.

19. Burns KEA, Adhikari NKJ, Meade MO: **Noninvasive positive pressure ventilation as a weaning strategy for intubated adults with respiratory failure**. *Cochrane Database of Systematic Reviews* 2004, **2**:2.

20. Carless PA, Henry DA, Moxey AJ, O'Connell D, McClelland B, Henderson KM, Sly K, Laupacis A, Fergusson D: **Desmopressin for minimising perioperative allogeneic blood transfusion**. *Cochrane Database Syst Rev* 2004(1):CD001884.

21. Carson JL, Hill S, Carless P, Hebert P, Henry D: **Transfusion triggers: a systematic review of the literature**. *Transfus Med Rev* 2002, **16**(3):187-199.

22. Carter BG, Butt W: **Review of the use of somatosensory evoked potentials in the prediction of outcome after severe brain injury**. *Crit Care Med* 2001, **29**(1):178-186.

23. Choi P-L, Yip G, Quinonez LG, Cook DJ: **Crystalloids vs. colloids in fluid resuscitation: A systematic review**. *Critical Care Medicine* 1999, **27**(1):200-210.

24. Claassen J, Hirsch LJ, Emerson RG, Mayer SA: **Treatment of refractory status epilepticus with pentobarbital, propofol, or midazolam: a systematic review**. *Epilepsia* 2002, **43**(2):146-153.

25. Cook D, Randolph A, Kernerman P, Cupido C, King D, Soukup C, Brun-Buisson C: **Central venous catheter replacement strategies: a systematic review of the literature**. *Crit Care Med* 1997, **25**(8):1417-1424.

26. Cook DJ, Reeve BK, Guyatt GH, Heyland DK, Griffith LE, Buckingham L, Tryba M: **Stress ulcer prophylaxis in critically ill patients. Resolving discordant meta-analyses.[see comment]**. *JAMA* 1996, **275**(4):308-314.

27. Cook DJ, Reeve BK, Scholes LC: **Histamine-2-receptor antagonists and antacids in the critically ill population: stress ulceration versus nosocomial pneumonia**. *Infect Control Hosp Epidemiol* 1994, **15**(7):437-442.

28. Corley DA, Cello JP, Adkisson W, Ko WF, Kerlikowske K: **Octreotide for acute esophageal variceal bleeding: a meta-analysis**. *Gastroenterology* 2001, **120**(4):946-954.

29. Cronin L, Cook DJ, Carlet J, Heyland DK, King D, Lansang MA, Fisher CJ, Jr.: **Corticosteroid treatment for sepsis: a critical appraisal and meta-analysis of the literature**. *Crit Care Med* 1995, **23**(8):1430-1439.

30. Curley MA: **Prone positioning of patients with acute respiratory distress syndrome: a systematic review**. *Am J Crit Care* 1999, **8**(6):397-405.

31. D'Amico G, Pietrosi G, Tarantino I, Pagliaro L: **Emergency sclerotherapy versus medical interventions for bleeding oesophageal varices in cirrhotic patients**. *Cochrane Database Syst Rev* 2002(1):CD002233.

32. D'Amico G, Pietrosi G, Tarantino I, Pagliaro L: **Emergency sclerotherapy versus vasoactive drugs for variceal bleeding in cirrhosis: a Cochrane meta-analysis**. *Gastroenterology* 2003, **124**(5):1277-1291.

33. D'Amico R, Pifferi S, Leonetti C, Torri V, Tinazzi A, Liberati A: **Effectiveness of antibiotic prophylaxis in critically ill adult patients: systematic review of randomised controlled trials.[see comment]**. *BMJ* 1998, **316**(7140):1275-1285.

34. De Franchis R: **Treatment of bleeding oesophageal varices: a meta-analysis**. *Scand J Gastroenterol Suppl* 1994, **207**:29-33.

35. de Jaeger A, Litalien C, Lacroix J, Guertin MC, Infante-Rivard C: **Protected specimen brush or bronchoalveolar lavage to diagnose bacterial nosocomial pneumonia in ventilated adults: a meta-analysis**. *Crit Care Med* 1999, **27**(11):2548-2560.

36. Dickinson K, Roberts I: **Medical anti-shock trousers (pneumatic anti-shock garments) for circulatory support in patients with trauma**. *Cochrane Database Syst Rev* 2000(2):CD001856.

37. Duley L, Henderson-Smart D: **Magnesium sulphate versus phenytoin for eclampsia**

**[Review]**. *Cochrane Database of Systematic Reviews* 2004, **2**:2.

38. Duley L, Henderson-Smart DJ: **Drugs for rapid treatment of very high blood pressure during pregnancy.[update in Cochrane Database Syst Rev. 2002;(4):CD001449; PMID: 12519557]**. *Cochrane Database of Systematic Reviews* 2000(2):CD001449.

39. Ebell MH, Becker LA, Barry HC, Hagen M: **Survival after in-hospital cardiopulmonary resuscitation. A meta-analysis**. *J Gen Intern Med* 1998, **13**(12):805-816.

40. Eichacker PQ, Gerstenberger EP, Banks SM, Cui X, Natanson C: **Meta-analysis of acute lung injury and acute respiratory distress syndrome trials testing low tidal volumes**. *Am J Respir Crit Care Med* 2002, **166**(11):1510-1514.

41. Eisele B, Lamy M, Thijs LG, Keinecke HO, Schuster HP, Matthias FR, Fourrier F, Heinrichs H, Delvos U: **Antithrombin III in patients with severe sepsis. A randomized, placeho-controlled, double-blind multicenter trial plus a meta-analysis on all randomized, placebo-controlled, double-blind trials with antithrombin III in severe sepsis**. *Intensive Care Medicine* 1998, **24**(7):663-672.

42. Faddy SC, Powell J, Craig JC: **Biphasic and monophasic shocks for transthoracic defibrillation: A meta analysis of randomised controlled trials**. *Resuscitation* 2003, **58**(1):9-16.

43. Fernandes HM, Gregson B, Siddique S, Mendelow AD: **Surgery in intracerebral hemorrhage. The uncertainty continues**. *Stroke* 2000, **31**(10):2511-2516.

44. Freeman BD, Isabella K, Lin N, Buchman TG: **A meta-analysis of prospective trials comparing percutaneous and surgical tracheostomy in critically ill patients.[see comment]**. *Chest* 2000, **118**(5):1412-1418.

45. Gadkary CA, Alderson P, Signorini DF: **Therapeutic hypothermia for head injury**. *Cochrane Database of Systematic Reviews* 2004, **2**:2.

46. Goulis J, Burroughs AK: **Role of vasoactive drugs in the treatment of bleeding oesophageal varices**. *Digestion* 1999, **60 Suppl 3**:25-34.

47. Hawkes CA, Dhileepan S, Foxcroft D: **Early extubation for adult cardiac surgical patients**. *Cochrane Database Syst Rev* 2003(4):CD003587.

48. Henderson WR, Dhingra VK, Chittock DR, Fenwick JC, Ronco JJ: **Hypothermia in the management of traumatic brain injury. A systematic review and meta-analysis**. *Intensive Care Med* 2003, **29**(10):1637-1644.

49. Heyland DK, Cook DJ, Jaeschke R, Griffith L, Lee HN, Guyatt GH: **Selective decontamination of the digestive tract. An overview**. *Chest* 1994, **105**(4):1221-1229.

50. Heyland DK, Cook DJ, King D, Kernerman P, Brun-Buisson C: **Maximizing oxygen delivery in critically ill patients: a methodologic appraisal of the evidence.[see comment]**. *Critical Care Medicine* 1996, **24**(3):517-524.

51. Heyland DK, Drover JW, Dhaliwal R, Greenwood J: **Optimizing the benefits and minimizing the risks of enteral nutrition in the critically ill: role of small bowel feeding.[see comment]**. *Jpen: Journal of Parenteral & Enteral Nutrition* 2002, **26**(6 Suppl):S51-55; discussion S56-57.

52. Heyland DK, Macdonald S, Keefe L, Drover JW: **Total parenteral nutrition in the critically III patient: A meta- analysis**. *Journal of the American Medical Association* 1998, **280**(23):2013-2019.

53. Heyland DK, Novak F, Drover JW, Jain M, Su X, Suchner U: **Should immunonutrition become routine in critically III patients? A systematic review of the evidence**. *Journal of the American Medical Association* 2001, **286**(8):944-953.

54. Heys SD, Walker LG, Smith I, Eremin O: **Enteral nutritional supplementation with key nutrients in patients with critical illness and cancer: a meta-analysis of randomized controlled clinical trials**. *Ann Surg* 1999, **229**(4):467-477.

55. Hill SR, Carless PA, Henry DA, Carson JL, Hebert PC, McClelland DBL, Henderson KM: **Transfusion thresholds and other strategies for guiding allogeneic red blood cell transfusion**. *Cochrane Database of Systematic Reviews* 2004, **2**:2.

56. Ho A-H, Lee A, Karmakar MK, Dion PW, Chung DC, Contardi LH: **Heliox vs air-oxygen mixtures for the treatment of patients with acute asthma: A systematic overview**. *Chest* 2003, **123**(3):882-890.

57. Hughes RAC, van der Meche FGA: **Corticosteroids for Guillain-Barre syndrome**. *Cochrane Database of Systematic Reviews* 2004, **2**:2.

58. Hurley JC: **Concordance of endotoxemia with gram-negative bacteremia in patients with gram-negative sepsis: a meta-analysis**. *J Clin Microbiol* 1994, **32**(9):2120-2127.

59. Hurley JC: **Prophylaxis with enteral antibiotics in ventilated patients: Selective decontamination or selective cross-infection?** *Antimicrobial Agents & Chemotherapy* 1995, **39**(4):941-947.

60. Imperiale TF, Teran JC, McCullough AJ: **A meta-analysis of somatostatin versus vasopressin in the management of acute esophageal variceal hemorrhage**. *Gastroenterology* 1995, **109**(4):1289-1294.

61. Ioannou G, Doust J, Rockey DC: **Terlipressin for acute esophageal variceal hemorrhage**. *Cochrane Database Syst Rev* 2003(1):CD002147.

62. Ioannou GN, Doust J, Rockey DC: **Systematic review: terlipressin in acute oesophageal variceal haemorrhage**. *Aliment Pharmacol Ther* 2003, **17**(1):53-64.

63. Ivanov R, Allen J, Calvin JE: **The incidence of major morbidity in critically ill patients managed with pulmonary artery catheters: a meta-analysis.[see comment]**. *Critical Care Medicine* 2000, **28**(3):615-619.

64. Ivanov RI, Allen J, Sandham JD, Calvin JE: **Pulmonary artery catheterization: a narrative and systematic critique of randomized controlled trials and recommendations for the future**. *New Horiz* 1997, **5**(3):268-276.

65. Keenan SP: **Use of ultrasound to place central lines**. *J Crit Care* 2002, **17**(2):126-137.

66. Keenan SP, Kernerman PD, Cook DJ, Martin CM, McCormack D, Sibbald WJ: **Effect of noninvasive positive pressure ventilation on mortality in patients admitted with acute respiratory failure: a meta-analysis**. *Crit Care Med* 1997, **25**(10):1685-1692.

67. Keenan SP, Sinuff T, Cook DJ, Hill NS: **Which Patients with Acute Exacerbation of Chronic Obstructive Pulmonary Disease Benefit from Noninvasive Positive-Pressure Ventilation? A Systematic Review of the Literature**. *Annals of Internal Medicine* 2003, **138**(11).

68. Kellum JA, Angus DC, Johnson JP, Leblanc M, Griffin M, Ramakrishnan N, Linde-Zwirble WT: **Continuous versus intermittent renal replacement therapy: a meta-analysis**. *Intensive Care Med* 2002, **28**(1):29-37.

69. Kellum JA, J MD: **Use of dopamine in acute renal failure: a meta-analysis**. *Crit Care Med* 2001, **29**(8):1526-1531.

70. Kern JW, Shoemaker WC: **Meta-analysis of hemodynamic optimization in high-risk patients**. *Crit Care Med* 2002, **30**(8):1686-1692.

71. Klerk CP, Smorenburg SM, Buller HR: **Thrombosis prophylaxis in patient populations with a central venous catheter: a systematic review.[see comment]**. *Archives of Internal Medicine* 2003, **163**(16):1913-1921.

72. Kollef MH: **The role of selective digestive tract decontamination on mortality and respiratory tract infections. A meta-analysis**. *Chest* 1994, **105**(4):1101-1108.

73. Krafft P, Fridrich P, Pernerstorfer T, Fitzgerald RD, Koc D, Schneider B, Hammerle AF, Steltzer H: **The acute respiratory distress syndrome: definitions, severity and clinical outcome. An analysis of 101 clinical investigations**. *Intensive Care Med* 1996, **22**(6):519-529.

74. Kwan I, Bunn F, Roberts I: **Timing and volume of fluid administration for patients with bleeding**. *Cochrane Database Syst Rev* 2003(3):CD002245.

75. Langham J, Goldfrad C, Teasdale G, Shaw D, Rowan K: **Calcium channel blockers for acute traumatic brain injury**. *Cochrane Database of Systematic Reviews* 2004, **2**:2.

76. Lefering R, Neugebauer EA: **Steroid controversy in sepsis and septic shock: a meta-analysis**. *Crit Care Med* 1995, **23**(7):1294-1303.

77. Li J: **Capnography alone is imperfect for endotracheal tube placement confirmation during emergency intubation**. *J Emerg Med* 2001, **20**(3):223-229.

78. Liberati A, D'Amico R, Pifferi S, Telaro E: **Antibiotic prophylaxis in intensive care units: meta-analyses versus clinical practice**. *Intensive Care Med* 2000, **26 Suppl 1**:S38-44.

79. Liberati A, D'Amico R, Pifferi Torri V, Brazzi L, Tinazzi A: **Antibiotic prophylaxis to reduce respiratory tract infections and mortality in adults receiving intensive care**. *Cochrane Database of Systematic Reviews* 2004, **2**:2.

80. Lightowler JV, Wedzicha JA, Elliott MW, Ram FSF: **Non-invasive positive pressure ventilation to treat respiratory failure resulting from exacerbations of chronic obstructive pulmonary disease: Cochrane systematic review and meta-analysis**. *British Medical Journal* 2003, **326**(7382):185-187.

81. Lysakowski C, Walder B, Costanza MC, Tramer MR: **Transcranial Doppler versus angiography in patients with vasospasm due to a ruptured cerebral aneurysm: A systematic review**. *Stroke* 2001, **32**(10):2292-2298.

82. Magarey JM: **Propofol or midazolam--which is best for the sedation of adult ventilated patients in intensive care units? A systematic review**. *Aust Crit Care* 2001, **14**(4):147-154.

83. Marik PE: **Low-dose dopamine: a systematic review**. *Intensive Care Med* 2002, **28**(7):877-883.

84. Marik PE, Zaloga GP: **Gastric versus post-pyloric feeding: a systematic review**. *Crit Care* 2003, **7**(3):R46-51.

85. Marik PE, Zaloga GP: **Early enteral nutrition in acutely ill patients: a systematic review.[see comment][erratum appears in Crit Care Med 2002 Mar;30(3):725]**. *Critical Care Medicine* 2001, **29**(12):2264-2270.

86. Markovitz BP, Randolph AG: **Corticosteroids for the prevention and treatment of post-extubation stridor in neonates, children and adults**. *Cochrane Database of Systematic Reviews* 2004, **2**:2.

87. Mauer DK, Nolan J, Plaisance P, Sitter H, Benoit H, Stiell IG, Sofianos E, Keiding N, Lurie KG: **Effect of active compression-decompression resuscitation (ACD-CPR) on survival: a combined analysis using individual patient data**. *Resuscitation* 1999, **41**(3):249-256.

88. McIntyre LA, Fergusson DA, Hebert PC, Moher D, Hutchison JS: **Prolonged therapeutic hypothermia after traumatic brain injury in adults: a systematic review**. *Jama* 2003, **289**(22):2992-2999.

89. Meade M, Guyatt G, Cook D, Griffith L, Sinuff T, Kergl C, Mancebo J, Esteban A, Epstein S: **Predicting success in weaning from mechanical ventilation**. *Chest* 2001, **120**(6 Suppl):400S-424S.

90. Meade M, Guyatt G, Sinuff T, Griffith L, Hand L, Toprani G, Cook DJ: **Trials comparing alternative weaning modes and discontinuation assessments**. *Chest* 2001, **120**(6 Suppl):425S-437S.

91. Meade MO, Guyatt G, Butler R, Elms B, Hand L, Ingram A, Griffith L, Cook DJ: **Trials comparing early vs late extubation following cardiovascular surgery**. *Chest* 2001, **120**(6 SUPPL).

92. Meade MO, Guyatt GH, Cook DJ, Sinuff T, Butler R: **Trials of corticosteroids to prevent postextubation airway complications**. *Chest* 2001, **120**(6 Suppl):464S-468S.

93. Messori A, Trippoli S, Vaiani M, Gorini M, Corrado A: **Bleeding and pneumonia in intensive care patients given ranitidine and sucralfate for prevention of stress ulcer: meta-analysis of randomised controlled trials.[see comment]**. *BMJ* 2000, **321**(7269):1103-1106.

94. Montejo JC, Zarazaga A, Lopez-Martinez J, Urrutia G, Roque M, Blesa AL, Celaya S, Conejero R, Galban C, Garcia de Lorenzo A *et al*: **Immunonutrition in the intensive care unit. A systematic review and consensus statement**. *Clin Nutr* 2003, **22**(3):221-233.

95. Nathens AB, Marshall JC: **Selective decontamination of the digestive tract in surgical patients: a systematic review of the evidence.[see comment]**. *Archives of Surgery* 1999, **134**(2):170-176.

96. Novak F, Heyland DK, Avenell A, Drover JW, Su X: **Glutamine supplementation in serious illness: a systematic review of the evidence.[see comment]**. *Critical Care Medicine* 2002, **30**(9):2022-2029.

97. Pang D, Keenan SP, Cook DJ, Sibbald WJ: **The effect of positive pressure airway support on mortality and the need for intubation in cardiogenic pulmonary edema: a systematic review**. *Chest* 1998, **114**(4):1185-1192.

98. Parameswaran K, Belda J, Rowe BH: **Addition of intravenous aminophylline to beta2-agonists in adults with acute asthma**. *Cochrane Database Syst Rev* 2000(4):CD002742.

99. Pascher A, Sauer IM, Hammer C, Gerlach JC, Neuhaus P: **Extracorporeal liver perfusion as hepatic assist in acute liver failure: A review of world experience**. *Xenotransplantation* 2002, **9**(5):309-324.

100. Peter JV, Moran JL, Phillips-Hughes J, Warn D: **Noninvasive ventilation in acute respiratory failure - A meta-analysis update**. *Critical Care Medicine* 2002, **30**(3):555-562.

101. Petrucci N, Iacovelli W: **Ventilation with lower tidal volumes versus traditional tidal volumes in adults for acute lung injury and acute respiratory distress syndrome**. *Cochrane Database of Systematic Reviews* 2004, **2**:2.

102. Platell C, Cooper D, Hall JC: **A meta-analysis of peritoneal lavage for acute pancreatitis**. *Journal of Gastroenterology & Hepatology* 2001, **16**(6):689-693.

103. Pronovost PJ, Angus DC, Dorman T, Robinson KA, Dremsizov TT, Young TL: **Physician staffing patterns and clinical outcomes in critically III patients: A systematic review**. *Journal of the American Medical Association* 2002, **288**(17):2151-2162.

104. Ram FS, Picot J, Lightowler J, Wedzicha JA: **Non-invasive positive pressure ventilation for treatment of respiratory failure due to exacerbations of chronic obstructive pulmonary disease**. *Cochrane Database Syst Rev* 2004(1):CD004104.

105. Ram FSF: **Green light for first-line intervention: Respiratory failure due to exacerbations of chronic obstructive pulmonary disease successfully treated using non-invasive positive pressure ventilation**. *Minerva Pneumologica* 2003, **42**(3):115-126.

106. Randolph AG, Cook DJ, Gonzales CA, Andrew M: **Benefit of heparin in peripheral venous and arterial catheters: systematic review and meta-analysis of randomised controlled trials**. *Bmj* 1998, **316**(7136):969-975.

107. Randolph AG, Cook DJ, Gonzales CA, Brun-Buisson C: **Tunneling short-term central venous catheters to prevent catheter-related infection: a meta-analysis of randomized, controlled trials.[see comment]**. *Critical Care Medicine* 1998, **26**(8):1452-1457.

108. Randolph AG, Cook DJ, Gonzales CA, Pribble CG: **Ultrasound guidance for placement of central venous catheters: a meta-analysis of the literature**. *Crit Care Med* 1996, **24**(12):2053-2058.

109. Raphael JC, Chevret S, Hughes RAC, Annane D: **Plasma exchange for Guillain-Barre syndrome**. *Cochrane Database of Systematic Reviews* 2004, **2**:2.

110. Roberts I: **Barbiturates for acute traumatic brain injury**. *Cochrane Database of Systematic Reviews* 2004, **2**:2.

111. Roberts I, Berger A: **Human albumin administration in critically ill patients: Systematic review of randomised controlled trials**. *British Medical Journal* 1998, **317**(7153):235-240.

112. Roberts I, Schierhout G, Alderson P: **Absence of evidence for the effectiveness of five interventions routinely used in the intensive care management of severe head injury: a systematic review.[see comment]**. *Journal of Neurology, Neurosurgery & Psychiatry* 1998, **65**(5):729-733.

113. Roberts I, Schierhout G, Wakai A: **Mannitol for acute traumatic brain injury**. *Cochrane Database of Systematic Reviews* 2004, **2**:2.

114. Rodrigo G, Pollack C, Rodrigo C, Rowe BH: **Heliox for nonintubated acute asthma patients**. *Cochrane Database of Systematic Reviews* 2004, **2**:2.

115. Rosenberg AL, Watts C: **Patients readmitted to ICUs: A systematic review of risk factors and outcomes**. *Chest* 2000, **118**(2):492-502.

116. Ruesch S, Walder B, Tramer MR: **Complications of central venous catheters: internal jugular versus subclavian access--a systematic review**. *Crit Care Med* 2002, **30**(2):454-460.

117. Scheer B, Perel A, Pfeiffer UJ: **Clinical review: complications and risk factors of peripheral arterial catheters used for haemodynamic monitoring in anaesthesia and intensive care medicine**. *Critical Care (London)* 2002, **6**(3):199-204.

118. Schierhout G, Roberts I: **Fluid resuscitation with colloid or crystalloid solutions in critically ill patients: a systematic review of randomised trials.[see comment]**. *BMJ* 1998, **316**(7136):961-964.

119. Silance PG, Vincent JL: **Oxygen extraction in patients with sepsis and heart failure: Another look at clinical studies**. *Clinical Intensive Care* 1994, **5**(1):4-14.

120. Silva CC, Saconato H, Atallah AN: **Metoclopramide for migration of naso-enteral tube**. *Cochrane Database Syst Rev* 2002(4):CD003353.

121. Schierhout G, Roberts I: **Hyperventilation therapy for acute traumatic brain injury**. *Cochrane Database Syst Rev* 2000(2):CD000566.

122. Sokol J, Jacobs SE, Bohn D: **Inhaled nitric oxide for acute hypoxemic respiratory failure in children and adults.[update of Cochrane Database Syst Rev. 2000;(4):CD002787; PMID: 11034763]**. *Cochrane Database of Systematic Reviews* 2003(1):CD002787.

123. Sokol J, Jacobs SE, Bohn D: **Inhaled nitric oxide for acute hypoxic respiratory failure in children and adults: a meta-analysis**. *Anesth Analg* 2003, **97**(4):989-998.

124. Steltzer H, Hiesmayr M, Mayer N, Krafft P, Hammerle AF: **The relationship between oxygen delivery and uptake in the critically ill: is there a critical or optimal therapeutic value? A meta-analysis**. *Anaesthesia* 1994, **49**(3):229-236.

125. Thackray S, Easthaugh J, Freemantle N, Cleland JG: **The effectiveness and relative effectiveness of intravenous inotropic drugs acting through the adrenergic pathway in patients with heart failure-a meta-regression analysis**. *Eur J Heart Fail* 2002, **4**(4):515-529.

126. Tonelli M, Manns B, Feller-Kopman D: **Acute renal failure in the intensive care unit: a systematic review of the impact of dialytic modality on mortality and renal recovery.[see comment]**. *American Journal of Kidney Diseases* 2002, **40**(5):875-885.

127. Travers AH, Rowe BH, Barker S, Jones A, Camargo CA, Jr.: **The effectiveness of IV beta-agonists in treating patients with acute asthma in the emergency department: a meta-analysis.[see comment]**. *Chest* 2002, **122**(4):1200-1207.

128. Treggiari MM, Walder B, Suter PM, Romand JA: **Systematic review of the prevention of delayed ischemic neurological deficits with hypertension, hypervolemia, and hemodilution therapy following subarachnoid hemorrhage**. *J Neurosurg* 2003, **98**(5):978-984.

129. Upchurch GR, Jr., Demling RH, Davies J, Gates JD, Knox JB: **Efficacy of subcutaneous heparin in prevention of venous thromboembolic events in trauma patients**. *Am Surg* 1995, **61**(9):749-755.

130. van de Beek D, de Gans J, McIntyre P, Prasad K: **Corticosteroids in acute bacterial meningitis**. *Cochrane Database of Systematic Reviews* 2004, **2**:2.

131. Vandycke C, Martens P: **High dose versus standard dose epinephrine in cardiac arrest - a meta-analysis**. *Resuscitation* 2000, **45**(3):161-166.

132. Villatoro E, Larvin M, Bassi C: **Antibiotic therapy for prophylaxis against infection of pancreatic necrosis in acute pancreatitis**. *Cochrane Database of Systematic Reviews* 2004, **2**:2.

133. Vincent JL, Dubois MJ, Navickis RJ, Wilkes MM: **Hypoalbuminemia in acute illness: is there a rationale for intervention? A meta-analysis of cohort studies and controlled trials**. *Ann Surg* 2003, **237**(3):319-334.

134. Wade C, Grady J, Kramer G: **Efficacy of hypertonic saline dextran (HSD) in patients with traumatic hypotension: meta-analysis of individual patient data**. *Acta Anaesthesiol Scand Suppl* 1997, **110**:77-79.

135. Wade CE, Kramer GC, Grady JJ, Fabian TC, Younes RN: **Efficacy of hypertonic 7.5% saline and 6% dextran-70 in treating trauma: A meta-analysis of controlled clinical studies**. *Surgery* 1997, **122**(3):609-616.

136. Walder B, Elia N, Henzi I, Romand JR, Tramer MR: **A lack of evidence of superiority of propofol versus midazolam for sedation in mechanically ventilated critically III patients: A qualitative and quantitative systematic review**. *Anesthesia & Analgesia* 2001, **92**(4):975-983.

137. Walder B, Pittet D, Tramer MR: **Prevention of bloodstream infections with central venous catheters treated with anti-infective agents depends on catheter type and insertion time: evidence from a meta-analysis**. *Infect Control Hosp Epidemiol* 2002, **23**(12):748-756.

138. Wijeysundera DN, Beattie WS, Rao V, Karski J: **Calcium antagonists reduce cardiovascular complications after cardiac surgery: A meta-analysis**. *Journal of the American College of Cardiology* 2003, **41**(9):1496-1505.

139. Yanagawa T, Bunn F, Roberts I, Wentz R, Pierro A: **Nutritional support for head-injured patients**. *Cochrane Database Syst Rev* 2002(3):CD001530.
